# Assessing the health impact of transnational corporations: a case study on McDonald’s Australia

**DOI:** 10.1186/s12992-016-0230-4

**Published:** 2017-02-06

**Authors:** Julia Anaf, Frances E. Baum, Matt Fisher, Elizabeth Harris, Sharon Friel

**Affiliations:** 1 Southgate Institute for Health, Society and Equity, Flinders University, GPO Box 2001, Adelaide, SA 5001 Australia; 2 Centre for Primary Health Care and Equity, University of New South Wales, Sydney,, 2052 Australia; 3 Regulatory Institutions Network, The Australian National University, Canberra,, ACT 2601 Australia

**Keywords:** Food industry, Globalization, Health equity, Transnational corporations

## Abstract

**Background:**

The practices of transnational corporations affect population health through production methods, shaping social determinants of health, or influencing the regulatory structures governing their activities. There has been limited research on community exposures to TNC policies and practices. Our pilot research used McDonald’s Australia to test methods for assessing the health impacts of one TNC within Australia.

**Methods:**

We adapted existing Health Impact Assessment methods to assess McDonald’s activities. Data identifying potential impacts were sourced through document analysis, including McDonald’s corporate literature; media analysis and semi-structured interviews. We commissioned a spatial and socioeconomic analysis of McDonald’s restaurants in Australia through Geographic Information System technology. The data was mapped against a corporate health impact assessment framework which included McDonald’s Australia’s political and business practices; products and marketing; workforce, social, environmental and economic conditions; and consumers’ health related behaviours.

**Results:**

We identified both positive and detrimental aspects of McDonald’s Australian operations across the scope of the CHIA framework. We found that McDonald’s outlets were slightly more likely to be located in areas of lower socioeconomic status. McDonald’s workplace conditions were found to be more favourable than those in many other countries which reflects compliance with Australian employment regulations. The breadth of findings revealed the need for governments to strengthen regulatory mechanisms that are conducive to health; the opportunity for McDonald’s to augment their corporate social responsibility initiatives and bolster reputational endorsement; and civil society actors to inform their advocacy towards health and equity outcomes from TNC operations.

**Conclusion:**

Our study indicates that undertaking a corporate health impact assessment is possible, with the different methods revealing sufficient information to realise that strong regulatory frameworks are need to help to avoid or to mediate negative health impacts.

## Background

### Introduction

The practices of transnational corporations (TNCs) affect population health through production methods, shaping social determinants of health, or influencing the regulatory structures governing their activities [1–3]. Described as ‘the primary movers and shapers of the global economy’ ([4] p. 177), with revenues now surpassing those of many national governments [5]; many TNCs wield increasing social, economic and political influence in the globalised market economy and within individual countries. TNCs can contribute to health inequities if health effects resulting from their products and practices have disproportionate adverse impacts on socially or economically disadvantaged populations; or if they provide greater health benefits to already better off groups [6]. In this paper, we test the applicability of a corporate health impact assessment (CHIA) framework to assess the health impacts of the operations of a transnational corporation (TNC) in one country.

Despite increasing recognition of the health implications arising from TNC practices [7], including in relation to non-communicable diseases (NCDs) [8], there has been a lack of focus on impact of community exposures to TNC policies and practices [9]. A growing body of research examines the practices of industry sectors; for example food and beverage [2, 7, 10], tobacco, pharmaceutical, and extractive industries [11–13]. Focussing on industry sectors can be instructive, but does not address the whole corporation as a ‘foundational, social institution that affects health’ ([7] p. 6). The corporation may be understood as a distal, macro-level social structure impacting population health. Reforms must address the entity as an institution, rather than by targeting only individual industries, corporations or products. This entails understanding the purpose of corporations and the historical factors granting these entities many of the rights of natural persons, or ‘corporate personhood’ [7, 14].

### TNC influences on health

TNC products and operations can support improvements in population health. A social determinants of health perspective holds that government and private sector policies and practices shape people’s cumulative exposure across the life course through social, economic, psychosocial and material pathways that protect health or cause disease [15]. This model explains how TNC operations can result in both positive and detrimental health impacts within a range of industry sectors including food and beverages, tobacco [11, 16], pharmaceuticals [17–19], and extractive industries [13, 20].

Positive impacts include TNC investment in host countries which can contribute to national economic growth and development through innovation, economies of scale, productivity gains, technology transfer, infrastructure provision, access to markets, and workforce capacity building [21]. Subsequent improvements in employment opportunities, working conditions, or access to education are likely to significantly benefit population health [22]. National taxation revenue from TNC operations potentially allows for expanded social or health services, or improved access to health-care technologies. Some TNCs are committed to corporate social responsibility (CSR) programs, whereby they assess their social, environmental or health impacts and benchmark these against their competitors (see, for example [23]). TNCs may also bolster shared value, or create economic value in a way that also creates value for society by addressing its needs and challenges [24]. This involves identifying and addressing social problems that intersect with business operations. Corporations may also attract skilled workers by demonstrating a level of ethical and environmental responsibility [25, 26].

Detrimental impacts from TNC products and operations in the fast food sector result from a range of issues arising from the acceleration of food science since the 1980s that has facilitated production of a wide range of cheap, palatable products [27]. At the same time, with economic globalisation, a number of studies implicate the growth of TNCs that manufacture, distribute and market these highly processed foods on a global scale, as a key factor driving the nutrition transition across many countries [10, 28, 29]. The evidence suggests that through their considerable market and political power these corporations can shape food systems in ways that alter the local availability, price, nutritional quality, desirability, and ultimately consumption of such foods [30–35]. Because highly processed foods tend to be energy dense and high in salt, fat and sugar, but low in micronutrients, their consumption has been linked to rising rates of obesity and NCDs globally [36, 37].

‘Fast food’ is easily prepared processed food served in snack bars and restaurants as a quick meal, or to be taken away. In 2016 McDonald's was the most valuable fast food brand in the world with an estimated brand value of about 88.65 billion U.S. dollars: the combined value of its main competitors Starbucks, Subway and KFC [38]. The availability of fast-food outlets and the price of fast food have been positively associated with obesity both nationally and globally [39–41]. Research also suggests a connection between childhood obesity and the location of fast food chain outlets; both in terms of proximity to schools and the level of outlet density [42].

## Methods

### Step 1: adapting HIA methods to assess TNC activity

#### Health impact assessment

HIA is a structured, action-oriented, and solution-focused approach for assessing and predicting positive and negative health impacts of policies, programs and projects. Conducting a HIA incorporates six steps: screening, scoping, identification, assessment, decision-making and recommendations, and evaluation and follow-up. HIA considers health effects within a population and identifies appropriate actions by which to manage them; including through the policy-making process [43]. HIAs have most commonly been applied to prospective assessment of impacts of upcoming policy or practice changes, but may be used retrospectively to analyse evidence on past events to help predict future impacts and to provide decision-making support.

Equity focused health impact assessment (EFHIA) is a particular form of HIA promoted by public health organisations internationally as a strategy to ensure that health equity is considered when developing policies, processes and plans [44, 45]. EFHIA identifies the different impacts on particular population groups and whether these are inequitable i.e. resulting from avoidable and preventable differences in social or economic conditions [46].

**Fig. 1:**
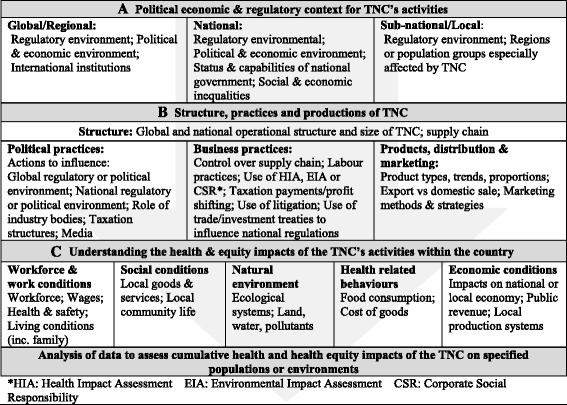
Corporate Health Impact Assessment Framework (CHIA)

A Corporate Health Impact Assessment (CHIA) Framework (Fig. 1) was developed to guide the process of adapting HIA methods to assess TNC activity [47]. It was devised during a meeting at the Rockefeller Foundation in Bellagio Italy in 2015, attended by 19 representatives from academia, the corporate sector, and civil society. A methodology had been lacking, and the meeting helped to identify ways to better understand and assess these health impacts on different communities [48].

The methods for the CHIA were also informed by and adapted from those in another retrospective HIA [49]. As with our study, this work drew upon the views of those directly affected as well as from expert opinion. Our research is the first of which we are aware on a TNC.

#### Selecting the TNC for the pilot study

Criteria were developed for choosing which industry sector and (subsequently) which corporation to assess; including consideration of the attributable burden of disease and the broad economic and social conditions under which the industry operates. McDonald’s [Australia] was selected as it is part of a large TNC serving greater levels of fast food than its combined competition [50]. This is facilitated by the level of marketing, incentives, and the suite of products including savoury and sweet products and sugary drinks. Importantly, McDonald’s is also the world’s largest franchisor, with more than 36,000 global outlets across 119 countries, and it employs 420,000 workers [51]. The pilot tested a range of methods with the aim to inform future research for assessing the health and health equity impacts of TNCs across a range of industry sectors and global and national contexts.

The identification of positive or negative health impacts of TNCs within the three parts of the CHIA framework has the potential to assist governments to devise appropriate regulatory mechanisms, and to provide corporations with insights for improving their corporate social responsibility, shared value commitments, and decision-making support. These issues are becoming increasingly important for corporate reputational endorsement and for benchmarking against competitors [52]. CHIA findings may also increase the evidence base for civil society actors and trade unions to inform their advocacy towards improving health and equity outcomes from TNC operations; by creating a demand for health-promoting regulatory and policy measures.

### Step 2. identification of potential impacts: data collection

Factors known to have positive and negative impacts on physical and mental population health outcomes and a range of relevant information were gathered from a number of sources and mapped against the CHIA framework. Data included documents, corporate literature, media items, semi-structured interviews focusing on McDonald’s Australian operations, and Geographic Information System (GIS) mapping [53]. During data collection some information was captured on other countries and McDonald’s global operations which contextualised Australian practices.

#### Documents

Documents including articles, government and parliamentary papers, NGO reports, online petitions, media releases and websites were accessed. A search was conducted of the Flinders University library and Scopus databases using the search term ‘McDonald’s Australia’ for the timeframe 2010–2015, and online searches using a wider range of search terms. These strategies sought to garner a broader range of material to add to that already gathered as part of a TNC literature review across several industry sectors. Ninety six items that included any relevant material for the CHIA framework were saved for review after employing the additional search terms. This broader review included material relating to McDonald’s USA which reports on Australian operations; and for identifying civil society campaigns in different Australian states.

#### McDonald’s Australia corporate literature

A search of McDonald’s Australia’s website and general web-related searches identified an additional 25 relevant items on McDonald’s products and operations which were saved for review. These included comprehensive product nutrition and energy information, action plans on marketing to children, information on occupational health and safety, employment and training, franchising, corporate social responsibility and sustainability programs, and corporate philanthropy. Most corporate literature was available on McDonald’s Australia’s website. This was augmented by specific searches on McDonald’s (USA) website to access annual reports and other Australian information which is not reported separately. This helped to put aspects of McDonald’s Australia’s operations into a global context.

#### Media items

To help identify the framing of news and media items relating to McDonald’s Australia a search was conducted of the Proquest Australia and New Zealand Newsstand database for the timeframe 2010–2015, using the search term ‘McDonald’s Australia’ limited to Australian media. This strategy sought to identify the scope and focus of interest on McDonald’s operations in Australia as reported in the media; especially any references to health impacts. The search included all forms of available media and produced 452 items. Over 250 items were immediately discarded as they were either incidental references, of marginal interest, or were duplicated coverage across the wide range of media sources. Fifty nine items that could help inform the research and add to the wider literature were saved for further analysis.

#### Geographic information system technology

Expert advice was commissioned for a spatial and socioeconomic analysis of the more than 900 McDonald’s restaurants. Australia has 2093 SA2s (second smallest spatial unit used by the Australian Bureau of Statistics) with an average population of 10,000 (range 3000–25,000). McDonald’s restaurants were matched to their SA2s to calculate the number of persons per outlet. This augmented the limited literature offering health equity insights on McDonald’s operations. Other evidence was provided in the form of economic statistics, maps and charts.

#### Semi- structured interviews

Interview participants were selected to gain diverse perspectives on McDonald’s Australia’s products and operations. Permission was sought from McDonald’s Australia to interview six or more senior executives who could assist the research in areas of corporate social responsibility, sustainability, philanthropy, employment, and corporate communications, but participation was denied. Ethics approval was subsequently obtained to interview former McDonald’s Australia executives and/or franchisees and current food industry executives to gain other business perspectives on McDonald’s operations. Invitations to participate were also sent to civil society actors monitoring different aspects of McDonald’s operations in Australia. These participants were identified by purposive and snowball sampling initially based on two high profile campaigns in South Australia and Victoria over recent years. Interview schedules were tailored to each interview cohort and were all designed to elicit responses in the areas of health impact identified in the CHIA framework.

All potential participants were emailed a personalised invitation, a Participant Information Sheet and Consent Form. Of the seventeen civil society participants approached eleven agreed to an interview and six did not respond. Seven of these eleven participants were currently, or had previously been involved in campaigns against proposed new McDonald’s outlets in Victoria, South Australia and Western Australia. One respondent was a former local government official, one a medical specialist with an interest in combatting childhood obesity, and two were academics with interests in addressing obesity and diabetes. We have treated the views of these individual campaigners as valuable evidence, even if not derived from an organised base. However, these are views based on certain predicted impacts, rather than reporting actual, social, psychological and economic impacts resulting from new outlet developments.

Seven former McDonald’s Australia executives and franchisees were approached via information in their LinkedIn profiles. One agreed to an interview, four declined and two did not respond. None of the six current food industry executives approached from two major industry sector groups agreed to participate. Five declined and one did not respond. All twelve interviews were conducted by telephone and transcribed by professional transcription services. Ethics approval to conduct the study was obtained from the Flinders University Social and Behavioural Research Ethics Committee (Project No.6785).

### Step 3: assessment of impacts

The documents, media items and transcribed interviews were all imported into NVivo qualitative data analysis software and coded against the CHIA framework. The coding framework was structured to mirror the three sections identified in Fig. 1: the global political economy and regulatory context of TNC operations (level A); TNC global and national corporate structure, practices and products (level B); and areas of health impact nationally (level C) [47]. The five areas of identified health impact (level C) were coded for both positive and adverse health impacts.

## Results

### Political economic and regulatory context for TNC activities: CHIA level A

Level A includes issues related to corporate global structure, the regulatory environment and taxation. Our research focused on Australia, but during the process it was recognised that inter-country comparisons are important; including for comparing different regulatory contexts. Any findings relevant to CHIA level A are reported under CHIA levels B and C.

### McDonald’s structure, practices and productions: CHIA level B

In this section we discuss our findings on McDonald’s corporate structure, political and business practices, and its products and marketing as identified under CHIA level B. Table 1 presents a summary of the structure, practices and production of McDonald’s Australia.

**Table 1:** McDonald’s structure, practices, products and marketing

McDonald’s corporate structure
McDonald’s global company is managed under distinct geographic segments. Australian operations are part of the Asia Pacific, Middle East and Africa (APMEA) segment.
McDonald’s political practices
• McDonald’s engages lobbyists, with corporate strategies designed to ensure the least restrictive regulatory environments.
• McDonald’s engages in strategic industry alliances that can help influence regulatory oversight and promote corporate interests over health and wellbeing. This includes the integrated and creative marketing directed to children and young people.
McDonald’s business practices
• McDonald’s range of corporate social responsibility initiatives can contribute to more environmentally sustainable corporate operations with potential for improved population health and welfare.
• McDonald’s alliance with dieticians may contribute to improving the composition of ultra-processed food.
• McDonald’s corporate philanthropy can contribute towards positive health and wellbeing.
• However, McDonald’s influence over government policy via lobbyists and industry representative may compromise obesity prevention.
• McDonald’s taxation strategies undermine governments’ ability to fund health and welfare policies including funding for corporate monitoring and regulation.
• Claims of limited community consultation on new outlet expansion raises concerns over the power imbalance between McDonald’s Australia and local communities.
McDonald’s products and marketing
Products
• McDonald’s menu has evolved to include a range of healthier options.
• However, many of McDonald’s food products are ultra-processed, high in kilojoules, fats, sugar and sodium. These can lead to obesity which carries an increased risk of diabetes, cancers, premature strokes and cardio-vascular disease, a shorter lifespan, and other health and psychological problems.
• Childhood obesity is associated with poor psychological and social wellbeing, poor self-esteem, bullying, anxiety, stigma and depression.
Marketing
• Voluntary advertising codes may help McDonald’s to review marketing strategies However, monitoring of compliance relies on public complaints.
• McDonald’s engages high profile media support which may help strengthen integrated marketing to children. This promotes brand choices linked to unhealthy food and childhood obesity.
• Marketing of McDonald’s purchase-driven donations and range of sponsorships promotes purchasing practices which may put corporate interests ahead of health.
• McDonald’s online ordering, drive through outlets, and home delivery all provide ease of access to unhealthy products.

### McDonald’s political practices

McDonald’s Australia is a member of the Australian Food and Grocery Council (AFGC). As such it has the capacity to influence the national regulatory environment. The AFGC is the leading national organisation representing Australia’s packaged food, drink and grocery products manufacturers. Its role is to lobby to ‘help shape a business environment that encourages the food and grocery products industry to grow and remain profitable’ [54]. The AFGC Quick Service Restaurant Initiative (QSRI) Forum members are the major fast food outlets in Australia. The QSRI Forum has developed a common framework for fast food companies to promote only healthier choices to children as part of Australia’s self-regulated system of advertising and marketing [55].

The limitations of QSRI self-regulation include that it only applies to a very narrow range of advertised foods and does not cover packages sold as “family meals”, despite the fact that they are designed to be consumed by children and their parents [56]. As encapsulated within an AFGC commissioned report [57], the AFGC’s vision of regulation is framed in a way that is in direct conflict with that of public health advocates. The report states that “the current regulatory stance is overly risk averse with a narrow focus on minimising risks to health and the environment” ([57] p. iv). It calls for a cut to the operating budget of regulators, and for granting corporate approvals as the default position.

Food industry documents identify that McDonald’s Australia is also a member of the Business Council of Australia (BCA) which “provides a forum for Australian business leaders to contribute directly to public policy debates”, with membership comprising the CEOs of Australia’s top companies [58]. McDonald’s CEO, Andrew Gregory, is a committee member of the BCA’s Labour Market, Skills and Education Committee. McDonald’s Australia also employs one of the biggest lobbying firms, Barton Deakin, which “helps business work more effectively with the Liberal National Coalition in Government and Opposition around the country” [59]. McDonald’s Australia’s formal links with business lobby groups serve to support a market rather than public health ethos in its operations.

A former franchisee highlighted McDonald’s potential influence over political and regulatory structures:

*If you have a company that employs probably the best part of 100,000 employees you have a lot of clout… So McDonald’s will have a fair level of push in decision-making across what affects their business.*

This is the view of one person only and is not publicly verifiable. Lobbying government is conducted by professional lobbyists.

### McDonald’s business practices

McDonald’s Australia engages in a range of business practices that may provide positive outcomes for the community. Its website states:

*We are committed to sustainable business practices and are determined to conduct our operations in a manner that does not compromise the ability of future generations to meet their needs* [51]*.*

McDonald’s has a range of corporate social responsibility (CSR) initiatives including corporate philanthropy. The corporation’s website gives a comprehensive overview of the scope of CSR activities which include devising healthier menu options in collaboration with accredited dieticians; working to maintain a sustainable supply chain; improving packaging and waste management; and undertaking animal health and welfare audits [26]. McDonald’s Australia reports that, as part of its corporate philanthropy initiatives, it funds all general and administrative costs of Ronald McDonald House Charities. This is to ensure that 100% of these publicly donated funds are used for their programs supporting sick children and their families.

McDonald’s global business is conducted in geographic segments with Australian operations being part of the Asia Pacific, Middle East and Africa (APMEA) segment [60]. This structure allows for taxation strategies that bolster corporate profitability. Under international taxation legal structures, transfer pricing between two of the same companies allows for distortions in the price of trade, or transfer ‘mispricing’; and for minimising taxation through reporting profits in tax havens [61]. No single authority necessarily sees the complete tax accounts of the TNC as a whole [62], and there is a lack of single country taxation reporting.

This can result in declining tax revenues from corporations; forcing governments to substitute other taxes, with a regressive impact on income distribution and cuts to public investment in health and other forms of social and economic infrastructure [63]. A 2015 report documents McDonald’s global and Australian taxation minimisation strategies [64]. Such corporate taxation measures, often undertaken through complex transactions that are facilitated by large global accounting firms [65], also reduce the capacity of countries to build strong public sectors that can develop cross-sector policy coherence for health [66].

### McDonald’s Australia’s products

McDonald’s website provides extensive material on the nutritional value, energy levels, and allergy-related information across its food and beverage range [67]. It describes many products with high levels of fat (including saturated fats), sugar, salt and preservatives. This comprehensive overview of products, together with information from documents and media items, provides a focal point for product evaluation as part of the CHIA. Links are identified between the growing access and availability of McDonald’s products and its home-delivery service. As a nutritionist argues:

*It just makes it easier for people to get food that is high in saturated fat and high in salt and it encourages people to have meals that lack in vegetables* [68].

### McDonald’s marketing strategies

McDonald’s engages in a range of creative and integrated marketing strategies for product promotion. It frames these within responsible marketing approaches to children:

*We have a genuine commitment to advertising appropriately and continue to review the research in relation to the impact of advertising on childhood obesity* [55].

The scope of marketing including to young people through integrated marketing strategies was noted across the qualitative data sets, including McDonald’s corporate literature [69]. Integrated Marketing Communication (IMC) is arguably the major communication development of the last two decades and a relatively new concept in corporate strategy [70]. As revealed on McDonald’s Australia website, IMC does not reflect a single initiative but a cumulative effect that works to persuade children and young people, in particular, to make particular brand choices [71].

A medical specialist argues that using a Ronald McDonald clown mascot as an “ambassador for health” in children’s hospitals deflects criticism from health-damaging products, as obesity has been identified as the most urgent health challenge facing paediatricians [72]. Children under eight years of age are cognitively incapable of understanding the commercial imperative of television advertising and are particularly vulnerable to its persuasive techniques [73]. Obese children have a 25–50% chance of becoming obese adults; increasing to 78% for older obese adolescents [74].

Consumer marketing documents also demonstrate how important cause-related marketing is to McDonald’s operations. This is described as “taking a brand and adding emotional character and empathy” ([75] p. 284). Consumers are more loyal to companies who act in a positive manner, but cause-related marketing involves a range of loyalty and incentive marketing tactics designed primarily to facilitate the corporation’s financial success [75].

McDonald’s Australia has also built strategic links to bolster its charitable purposes, including through Ronald McDonald House Charities, Victoria Police, Vinnies CEO Sleep Out, Clean up Australia Day, and Earth Hour. This allows for an ascribed ‘halo effect’ when a company that markets unhealthy products tries not only to look good, but simultaneously seeks to distract from its unhealthy image. This is achieved by promoting positive images of its products and operations through its support for ‘worthy’ causes. As the burden of obesity falls most substantially upon disadvantaged communities [72], this is an important health equity impact.

### Understanding the health and equity impacts of McDonald’s activities within the country: (CHIA level C)

In this section we review positive and negative aspects relating to health and/or health equity across five domains: 1) McDonald’s workforce and working conditions, 2) social conditions, 3) environmental conditions, 4) economic conditions, and 5) health-related behaviours. A summary of our findings concerning actual or potential health and equity impacts across these five domains are summarised in in Table 2.

**Table 2:** Health and equity impacts of McDonald’s operations

Work and workforce conditions
• McDonald’s invests heavily in employment and training, is strongly committed to an inclusive workplace and occupational health and safety standards.
• McDonald’s is a respected national training provider and provides high level youth employment. However, McDonald’s does not pay penalty rates and many jobs are filled by casual and part-time workers with the low-levels of unionisation across the fast food industry.
Social conditions
• McDonald’s provides a low cost option for financially struggling families and a venue for inexpensive social interaction.
• However, location near schools has potential impacts on easy access to unhealthy food options and childhood obesity.
• Concerns have been raised over the negative impacts on housing prices in the vicinity of McDonald’s new outlets, and over impacts on local cafes and other services due to the comparative size and scale of McDonald’s operations.
• Negative health impacts reported include physical and psychological effects from community efforts to stop the proliferation of new McDonald’s outlets.
Environmental conditions
• McDonald’s ‘Five Pillars’ sustainability framework is a positive initiative.
• However, resource-intensive operations would impact on global climate change both directly and indirectly, with externalisation of costs to the community.
• High level littering, food wastage, and impact on social amenity are other negative aspects.
• There is potential for ‘greenwash’ as part of corporate relations strategies and its links to community group abatement projects.
Economic conditions
• McDonald’s provides positive impacts from employment; including in their outlets and supply chains and from construction and infrastructure provision.
• Franchises provide positive economic benefits through a proven business model.
• However, there is externalisation of costs to the public from profit shifting, tax havens, and service fees paid back to USA headquarters.
• The health costs of non-communicable diseases and environmental impacts from McDonald’s operations are externalised to the community.
Health related behaviours
• Consumption of McDonald’s cheap and palatable but ultra-processed food and sugary drinks can contribute to increased levels of overweight and obesity, which is negatively correlated with socio-economic status.
• There is a link between consumption and McDonald’s sophisticated and integrated marketing strategies, including from the influence of the ‘halo effect’ and ‘health washing’.
• Bundled products, drive through outlets and home delivery also influence consumption patterns.
• There is an association between consumption of McDonald’s products, lower socio-economic status, and children and young adults; with implications for health equity

#### Workforce and working conditions

The qualitative data sets all contributed to informing the CHIA in relation to McDonald’s Australia’s employment conditions. They provided incidental information on comparative wages for McDonald’s workers across a range of countries which contextualises Australian workforce issues.

Employment is a critical determinant of health providing both financial and non-material benefits [76]. McDonald’s corporate literature, documents from the Fair Work Commission, the Fair Work Ombudsman and several media items informed the CHIA on a range of positive aspects of McDonald’s workforce and working conditions. McDonald’s Australia’s formal employment agreement provides benefits to workers by regulating wages and conditions. These are negotiated between the Fair Work Commission, Australia’s national workplace relations tribunal, and the Shop Distributive and Allied Employees Association Union and provide a level of job security for the corporation’s Australian employees [77].

We collected information on workforce age and salary levels, and high level youth employment, offering the benefits of a first job [77]. McDonald’s Australia provides employment to 90,000 individuals and spends over $1billion annually on wages, salaries and bonuses [26]. McDonald’s own corporate literature and media items also highlight its large Australian investment in employment and high quality workforce training as an Accredited Training Provider for Certificate level qualifications and Diplomas and Advanced Diplomas of Management.

Unemployment is one of the constellation of disadvantages experienced by Aboriginal people [78] and McDonald’s is committed to developing structures supporting Indigenous Australian workers; as well as people living with disabilities. McDonald’s literature and a newspaper article note the corporation’s inclusive, non-discriminatory workplace with a high percentage of women in leadership roles. Fifty six per cent of management positions are held by women, which far exceeds the eight per cent of senior female leadership roles in ASX 200 companies [79].

Occupational illness and injury is a critical public health issue due to the pain and suffering of individual workers and economic costs to workers, employers and the broader community [78]. McDonald’s website explains its commitment to, and ongoing improvement of, occupational health and safety measures through monitoring Lost Time Injury Frequency Rates (LTIFR) and its compliance with the law [80]. The Fair Work Ombudsman endorses McDonald’s Australia for providing “an opportunity for young people to learn skills that they can apply throughout their working lives” [81]*.* This positive view was also endorsed by a respondent who was a former McDonald’s franchisee.

However, most young people who work in McDonald’s are engaged in lower skill activities. One civil society actor opposing the proposal for a new McDonald’s outlet provided a different perspective on the mainly casual employment offered by McDonald’s:

*…look they always claim they offer 60, 80 or 100 jobs. What they don't say that it’s rarely full-time jobs, there's really only a handful of full-time jobs at every McDonald's outlet, other than that, it's school kids or students that work a few hours here and there for $9 an hour.*

Other contested views on McDonald’s Australia’s employment conditions were expressed in documents, media items and interviews which reported variously on specific, albeit limited, incidents in relation to staff safety issues, food production practices, occupational health and safety incidents; and the fact that McDonald’s does not pay penalty rates [82]. This form of remuneration mediates precarious employment: one of the health-damaging features of the growing increase in non-standard forms of work which includes the job insecurity of low paying and often insufficient work.

The Fair Work Ombudsman confirms that most of McDonald’s Australia’s 90,000 workers are casual [83]. Casualization is one facet of precarious employment, with casual workers lacking job security beyond a particular shift [84]. Low-level unionisation across the fast food industry more generally is characterised by high levels of casual and part-time workers who are typically young and poorly informed about workplace rights, and by high staff turnover. Even with these constraints Australian regulations on wages and conditions covering McDonald’s Australia’s workers are more protective in comparison with some other jurisdictions with less stringent regulations; or compared with being unemployed.

#### Social conditions

Recently there has been greater acknowledgement of the role that the built environment plays in influencing healthy outcomes related to dietary choices [85]. This aspect of the health impact framework relates to the positive and negative impacts of McDonald’s operations on local community life and on local goods and services. McDonald’s own literature presents the ways in which the corporation interacts with and supports local communities through a range of sponsorships and corporate philanthropy initiatives. Mc Donald’s website states that as one of its seven core values:

*We give back to our communities We look after the locals*

*We take seriously the responsibilities that come with being a leader. We help our customers build better communities, support RMHC®, and leverage our size, scope and resources to help make the world a better place* [55]*.*

McDonald’s does not directly address its benefits to social life, but images of happy families at their restaurants in their advertising material suggest they like to represent the restaurants as a convivial place for family gatherings. Their website highlights a range of family activities, including catering for children’s birthday parties and a range of other in-house and online games and playground equipment. McDonald’s remains a low-cost option for financially struggling families and a “fun” environment for children; especially those whose families have fewer options.

### Civil society activism against McDonald’s impact on local communities

Positive social impacts were countered, unsurprisingly, by civil society activists we spoke to; highlighting the negative effects of McDonald’s on their local communities. The analogy of ‘David vs Goliath’ in portraying the perceived power disparity between McDonald’s and the various local communities was a recurring theme.

#### Stress and anxiety

Activists noted that engaging in civil society protests against McDonald’s could have a negative impact on their mental well-being which led to stress and anxiety resulting from perceived loss of control. In the Supreme Court McDonald’s sought unspecified damages and costs and wide-ranging injunctions against protesters at Tecoma Victoria for either remaining on or entering the site and for using social media to prosecute their campaign [86, 87]. One campaigner against the expansion of McDonald’s outlets argued:

*The problem is McDonalds have got very deep pockets and a lot of money, so that scares us… we don't have the funds to fight… it feels like a big David and Goliath battle, because they have got powerful lawyers and deep pockets, and they'll keep pushing, pushing.*

Stress due to potential litigation by McDonald’s was noted by another activist:

*When you’re dragged into the Supreme Court with the prospect of losing your house you begin to wonder whether you’ve taken on the wrong enemy.*

One Western Australian campaigner spoke of the personal experience of the stress-related toll on campaigners who were often local residents:

*I find the whole process very stressful. So mental health is an issue just as much as physical health. The fear of the unknown is stressful.*

#### Economic impacts

Another activist noted economic-related stress:

*Aside from all this, there is a known impact to property prices… So that is a stress to people.*

We found that activists also had a fear of the economic impact of strategic lawsuits against public protest (‘SLAPP’ suits) [2], and these various accounts support Freudenberg’s (2014) argument of a power imbalance between McDonald’s and local communities in respect of litigation. An interviewee also spoke of the potential economic impact that McDonald’s outlets may have on local goods and services:

*It’s a cafe area and small business area and we’ve got a number of wonderful restaurants so that McDonald’s ability to impact on the income of those businesses was also of great concern.*

The power disparity between McDonald’s Australia and the local communities in which it seeks to embed its operations highlights important impacts on health and equity.

### Environmental conditions

Environmental conditions considered as part of the CHIA relate to impacts on the natural environment, including on ecological systems, land and water; pollution and loss of amenity. McDonald’s corporate literature indicates many positive environmental sustainability measures. The corporation has adopted the Five Pillars Sustainability Framework as part of its CSR initiatives. This framework covers corporate, restaurant and sustainable operations, advocacy and partnerships, and culture and communications. Environmental themes are included under each of the Five Pillars [26]. McDonald’s explains:

*We have an environment policy in place that underpins all of our decisions and actions… we operate within a sustainability framework which is designed to assist the entire McDonald’s business to define and deliver appropriate initiatives against the major environmental themes of five identified sustainability pillars* [51].

McDonald’s website also highlights a range of initiatives including energy reduction, pollution abatement and ‘clean streets’ campaigns which ameliorate negative environmental impacts.

McDonald’s Australia’s website also notes other pollution abatement strategies including organic waste collection, and conversion of used cooking oil to biodiesel fuel for delivery trucks in Victoria. McDonald’s has also been the recipient of packaging awards from the Australian Packaging Covenant for demonstrating a commitment to environmental sustainability and efforts to minimise waste [88]. It uses Rainforest Alliance certified coffee produced under standards intended to improve health by protecting the environment and the rights of workers in other countries [89].

Other data highlighted a different perspective on McDonald’s environmental impacts; including potential “greenwashing” or cleaning the corporate image by showing concern for the planet and welfare of all as part of a corporate relations strategy [90]. McDonald’s message that reducing and taking responsibility for waste and environmental degradation by supporting “green” groups including Clean Up Australia and Earth Hour is arguably a reactive stance; conflicting with corporate business operations that prioritise profit making and increased growth and consumption [91].

Less visible negative environmental aspects include the impacts from transportation, refrigeration, and carbon and ecological footprints. These impact on global climate change; including the direct risks of respiratory illness, thermal extremes, natural disasters, ozone layer depletion; and the indirect effects of communicable diseases and food security [92]. McDonald’s operations also add to resource intensive meat and dairy production and animal husbandry under which 70% of global agricultural land is appropriated for animal rearing. This leads to soil erosion, freshwater pollution, exorbitant water use, high pesticide levels, and sediments threatening aquatic environments [93], and contributes to global warming.

The Australian 2011/2012 National Branded Litter Study cited McDonald’s as the highest proportion of all litter items recorded across Australia (12.8%, up from 9.85% in 2007/2008) [94]. Litter is an environmental crime, causing harm to animal and marine life, blockages to storm water system and flooding. Litter was a major environmental issue for civil society actors opposed to proposed new McDonald’s outlets, especially in tourism areas. One respondent argued:

*They’re the number one brand litter source in Australia, McDonald’s. One in every eight pieces of litter bears McDonald’s brand and I can’t help but believe that that the heavy branding on all of their products…is sort of a secondary avenue of advertising.*

A former franchisee outlined McDonald’s imperative for adopting their range of litter abatement strategies:

*They want to be able to tell a story: “Well, McDonald’s Australia doesn’t litter, unfortunately the consumers do, and it’s their way of saying we’re supporting that by doing biodegradable products and supporting activities like Clean up Australia Day”.*

From a health equity perspective, the negative impacts from littering must also be understood within the context of greater numbers of fast food outlets being situated in lower socio-economic areas. Massive food wastage [95] impacts on natural resources, compromises sustainable development, and has implications for intergenerational inequity [96].

### Economic conditions

This aspect of CHIA level C refers to the impact on economic conditions from McDonald’s Australia’s influence on the national or local economy, local supply and purchasing systems, and employment. McDonald’s Australia’s own business profile provides information on the level of value-adding from their operations, the number of jobs created with each new outlet, high level support for local producers, and its contribution to Australia’s gross domestic product (GDP). As well as being a large employment provider, McDonald’s sources products from 9000 Australian suppliers, invests more than $40 million every year in training crew, restaurant managers and corporate staff, and supporting the administrative operation of the publicly-funded Ronald McDonald House Charities [97]. A former McDonald’s franchisee explains that there are positive economic impacts accruing from the corporation’s infrastructure upgrades, such as those to promote sustainable watering systems for outlet landscaping.

These positive economic factors are mediated by actual and potential loss of state revenue through business profit shifting as part of McDonald’s taxation strategy. A 2015 international report provided important insights into McDonald’s taxation strategies both globally and nationally, and contextualised Australian taxation arrangements within its global operations:

*McDonald’s uses royalty payments from franchisees and foreign subsidiaries in major markets to route profits to tax havens. These strategies may have allowed it to avoid up to US$1.8 billion in tax in those markets in the years between 2009 and 2013, including €1 billion across Europe and AU$497 million in Australia* [98].

The McDonald’s material we examined showed that tax minimization, through the way the company is structured, was part of their Australian business model and profit seeking as outlined in an Australian case study included in a report on McDonald’s international taxation strategies [64].

Corporations including McDonald’s may also cause negative economic and associated health impacts to the extent that legal corporate entities are able to externalize, i.e. shift to consumers, taxpayers, or society as a whole, the real costs of production or consumption of their products. The economic impact of the cost of externalities produced by McDonald’s include the cost burden of chronic disease and dealing with environmental waste. As corporations are not required to pay such costs, the public subsidizes increased profits, which in turn leads to increased production; magnifying the adverse impact on population health [99]. The disease and cost burdens associated with obesity, overweight and chronic diseases in 2008 in Australia were calculated to be $58.2 billion; comprising $8.3 billion in direct financial losses and $49.9 billion in net costs of lost wellbeing [100].

### Health-related behaviours

The Australian Bureau of Statistics reports that in 2014–2015 63.4% of Australian adults and 27.4% of children were overweight or obese [101]. McDonald’s website states its responsibility towards public health:

*We’re concerned about issues such as obesity, improving our diets, our own health and the health of our children. McDonald’s Australia believes that as the country’s largest and best known name in the Quick Service Restaurant industry, it has a responsibility to take a leadership role in regard to the issue of public health in this country* [102].

Daube notes, however, that the food industry is under pressure from shareholders and others to act aggressively in the pursuit of profit [103]. For the population to lose weight, companies must sell less food containing high fat and high sugar. This pits the fundamental purpose of the food industry against public health goals [104]. Despite some positive changes to their products McDonald’s website shows that they still offer many ultra-processed and energy dense products with high levels of sugar, salt and fat [67].

However, as McDonald’s products are always a part of an overall diet, it is difficult to make a causal connection, but there is an association between growth in fast foods and population obesity in Australia, and many commentators link the two. A Melbourne Australia study suggested that socio-economic status and environmental determinants (density of fast food outlets) interact to create environments in which poorer people have increased exposure to energy dense foods [105]. Australian research on choices of fast food restaurants found that people accessing McDonald’s are often those who are travelling or “out and about”. It suggested that this may be due in part to the convenience of the high numbers of outlets, but also a perception that this was the only option for many people [106]. As one respondent maintained:

*The more affluent families tend to cook better foods at home and the less affluent tend to get a McDonald’s meal, or similar, because it’s cheap and it’s accessible… If you’re a struggling mum you can go to McDonald’s and get a meal for a toddler for $2…You’re probably thinking “I’ve put food in my child’s belly” and that’s really sad….*

The National Health Performance Agency notes that the six suburbs in Western Sydney to which McDonald’s home delivers has the second highest number of overweight or obese adults [107]. Media items flagged McDonald’s strategy of trialling its home delivery service in one of the most disadvantaged areas in Sydney, with one of the highest levels of obesity [108]. Other issues arising in this CHIA impact area related to food consumption and health-related behaviours are that “drive throughs” target ease of consumption; and that bundled foods are sold more cheaply.

#### Spatial distribution of access to outlets – implications for health equity

To inform the CHIA a spatial and socioeconomic analysis of McDonald’s restaurants in Australia was conducted using Geographic Information System (GIS) technology at the Statistical Area 2 level (SA2s) using the ARCMap facility in ARC GIS and CartoDB [53]. SA2s are medium sized general purpose spatial units constructed by the Australian Bureau of Statistics (ABS) representing a community that interacts together socially and economically [109]. The number of persons per McDonald’s restaurant for Socio Economic Index for Areas (SEIFA) quartiles using the Index of Relative Socio-economic Disadvantage (IRSD) are reported in Table 3.

**Table 3:** Number of persons per McDonald’s outlet by age group and quartile of SEIFA score (IRSD)

Quartile (Number of SA2s) Low-High	Number of McDonald’s outlets in Quartile	Number of persons per McDonalds’s outlet			
		Total population (all SA2s)	0–9 years	10–19 years	20–34 years
1 (518)	265	19,197	2,559	2,531	3,772
2 (516)	266	19,764	2,507	2,542	4,018
3 (526)	217	24,887	3,178	3,136	5,330
4 (539)	176	32,443	4,126	4,192	6,659
Total (2099)	924	23,220	2,988	2,993	4,759

McDonald’s outlets were slightly more likely to be located in areas of lower socioeconomic status. The socio-economic data presented in the table also identify the main consumer age groups from a fast food marketing and public health perspective - children (0-9 and 10-19) and young adults aged 20-34 years. The distribution shows that the bottom half of the socioeconomic distribution has fewer children per restaurant; in other words there is more market penetration in the lower socioeconomic quartiles [110]. The difference between the bottom and top quartiles is most stark; especially for the 20–34 age group. The mean SEIFA (IRSD) score for Australian SA2s which have a McDonalds restaurant is 988 (st dev = 70.7, *n* = 924). The mean SEIFA score for SA2s without McDonalds restaurants is 1002 (st dev = 86.6, *n* = 1495). This difference is statistically significant (t = 4.3, *p* < 0001), suggesting an inverse relationship between the location of McDonalds outlets and socioeconomic status.

## Discussion

This paper set out to answer a question on the extent to which it is possible to document the health effects of a transnational corporation in one country, Australia, using a CHIA framework and McDonald’s as the pilot. Actual and potential positive health outcomes are identified in McDonald’s investment in high levels of employment and training, and its inclusive, non-discriminatory workplaces. McDonald’s Australia’s workforce conditions, bolstered by Australian employment regulations, guarantee a level of worker protection that remains unavailable in some other jurisdictions. McDonald’s outlets also provide many opportunities for affordable social interaction; especially for people with fewer options. The corporation engages in CSR, shared value and philanthropic initiatives that provide public benefits.

However, there are potential negative health impacts from McDonald’s ultra-processed food; its strategic industry alliances that facilitate corporate influence over food and advertising regulation; the loss of state revenue from its taxation strategies; and its health and environmental costs that are externalised to the community. Other detrimental impacts relate to the power disparity between McDonald’s Australia and the local communities who oppose more fast food outlets. Although the corporation provides high level employment and training for younger age groups this is on lower wages and less secure conditions. It therefore reveals a mixed picture of employment in respect of the CHIA, and highlights a challenge for countries seeking increased employment opportunities, including for young people, without bearing the negative imposts from unhealthy food. On balance, positive impacts are outweighed by the negative impacts, including the potential contribution to high levels of obesity and chronic disease, the impacts of aggressive marketing to children and young people, and financial strategies resulting in a significant loss of revenue for health and social infrastructure.

The CHIA revealed regulatory shortcomings in respect of self-regulation governing advertising and marketing, especially to children and young people. It identified constraints in local planning in different Australian jurisdictions, and taxation structures that are enabling for McDonald’s. The implications from findings at all levels of the CHIA are the need for rigorous national and supra-national policies [48]. Voluntary codes for TNC operations have little public accountability, are difficult to reinforce other than by negative publicity, and divert attention away from legal compliance [7]. Realistic policies will go beyond information and education to include fiscal and statutory constraints [27]. To mediate negative health impacts, different marketing approaches are also needed, food must be healthier, and efforts made to better address the corporation’s environmental footprint.

One limitation of the research was the decision of McDonald’s Australia and the industry sector to not participate in the interviews which would have provided more nuanced perspectives of the information included in their corporate literature. This is a challenge, and alternative strategies are needed; for example, having corporate actors on the research team with specified roles. While the refusal of McDonald’s and other industry representatives to participate was limiting, it was still possible to gain a corporate view from publicly available sources. There are many evidence gaps on equity impacts, and social impacts that rely on the perceptions of local actors prior to new McDonald’s outlets being established, even though these raise serious concerns. The research was designed to include a range of specific search terms. The use of an alternative strategy may have identified further information to support the postulated potential social impacts which are not verified in the data.

There are also a number of assumptions that have not been made explicit but underlie the structure of the study. Firstly, there are no clear links between consumption of McDonald’s products, chronic disease, and obesity and other health problems, although this seems likely. There is also an assumption that most fast food is consumed through major chains with little attention to the role of the many local food fish and chip, pizza and hamburger shops that are often closer and more convenient for people; especially for those without cars. It must also be acknowledged that a large proportion of sugary drinks are purchased in supermarkets for home consumption; not only from fast food outlets.

The CHIA is not a regulatory tool that is required in order to act; there was no specific policy, proposal or decision point it was trying to influence. It was not a community led process, nor was it clearly an advocacy process but this seems to be its most likely use. This in turn meant that processes for engagement, governance structures, weighting of evidence and processes for managing conflict or disagreement did not need to be in place. The process issues of conducting potentially contested findings could not be addressed/explored.

The lessons we have learned about challenges for conducting a CHIA include recognising the need to study a TNC globally by exploring its operations internationally, nationally, and locally. This allows for comparing practices (e.g. wage levels) across different jurisdictions. We identified how important this is but recognise that it would require much larger budgets and major research grants. This pilot study confirmed the feasibility and usefulness of the CHIA framework in gathering and assessing impacts of TNCs on health in a structured and systematic way.

## Conclusions

This paper demonstrates that it is possible to identify potential health impacts of policies, plans, projects and services related to TNCs; political economic and regulatory contexts for TNC’s activities; the structure, practices and productions of TNCs; and understanding the health and equity impacts of the TNC’s activities within the country. Our study indicates that undertaking a CHIA is possible, with the different methods revealing sufficient information to realise that strong regulatory frameworks are needed help to avoid or to mediate negative health impacts.

## Abbreviations

AFGC: Australian food and grocery council
BCA: Business council of australia
CHIA: Corporate health impact assessment
CSR: Corporate social responsibility
EFHIA: Equity focused health impact assessment
GDP: Gross domestic product
GIS: Geographic information system
HIA: Health impact assessment
IMC: Integrated marketing communication
IRSD: Index of relative socio-economic advantage
LTIFR: Lost time injury frequency rates
NCD: Non-communicable disease
QSR: Quick service restaurant
SA2: Statistical area level 2
SEIFA: Socioeconomic indexes for areas
